# Polarization toward Tfh2 cell involved in development of MBC and antibody responses against *Plasmodium vivax* infection

**DOI:** 10.1371/journal.pntd.0012625

**Published:** 2024-10-30

**Authors:** Pongsakorn Thawornpan, Zulfa Zahra Salsabila, Piyawan Kochayoo, Tipanan Khunsri, Chayapat Malee, Kittikorn Wangriatisak, Chaniya Leepiyasakulchai, Francis Babila Ntumngia, John H. Adams, Patchanee Chootong

**Affiliations:** 1 Department of Clinical Microbiology and Applied Technology, Faculty of Medical Technology, Mahidol University, Bangkok, Thailand; 2 Center for Global Health and Interdisciplinary Research, College of Public Health, University of South Florida, Tampa, Florida, United States of America; Centro de Pesquisa Gonçalo Moniz-FIOCRUZ/BA, BRAZIL

## Abstract

**Background:**

*Plasmodium vivax* is the dominant *Plasmodium* spp. causing malaria throughout tropical and sub**-**tropical countries. Humoral immunity is induced during *P*. *vivax* infection. However, data on longevity of antibody and memory B cell (MBC) responses is lacking. Follicular helper T cells (Tfh) are drivers of high**-**affinity and long**-**lived antibody responses. Understanding of Tfh**-**mediated immunity against malaria is valuable for vaccine development.

**Methodology/Principal findings:**

We enrolled 31 acutely infected *P*. *vivax* patients in low malaria transmission areas of Thailand to detect frequencies, phenotypes and kinetics of different subsets of circulating Tfh (cTfh) and MBCs, and to evaluate their association with humoral immunity following natural *P*. *vivax* infection. Expansion of cTfh2 cells, activated and atypical MBCs were shown during acute malaria. To relate increased cTfh2 cells to humoral immunity, *P*. *vivax***-**specific MBCs and antibodies were assessed. High anti**-**PvCSP and **-**PvDBPII seropositivity was detected and most subjects produced MBCs specific to these antigens. The increased cTfh2 cells were positively related to atypical MBCs, plasmablasts**/**plasma cells, and anti**-**PvDBPII IgM and IgG levels. Distributions of memory cTfh cell subsets were altered from central memory (CM) to effector memory (EM) during infection. The highest ratios of cTfh**-**EM**/**cTfh**-**CM were represented in cTfh2 cells. Positive correlation of cTfh17**-**EM with activated and atypical MBCs was observed, while cTfh2**-**CM and cTfh17**-**CM cells were positively related to PvDBPII**-**specific MBCs and IgM levels.

**Conclusions/Significance:**

Present study demonstrated that *P*. *vivax* infection induced cTfh polarization into cTfh2 subset, and alteration of memory cTfh2 phenotype from CM to EM phase. These *P*. *vivax***-**induced cTfh responses significantly associated with generation of MBCs and antibody responses. Therefore, cTfh2 cells might possibly influence humoral immunity by inducing expansion of activated and atypical MBCs, and by generating *P*. *vivax***-**specific MBCs and antibody responses following natural infection.

## Author summary

Naturally acquired immunity to malaria relies on the development of MBCs and long**-**lived plasma cells that serve as the source of antibody responses that inhibit parasite invasion. To date, information is limited regarding the role of Tfh cells in supporting development of MBCs and antibodies against *P*. *vivax* infection, particularly in the context of natural malaria exposure. Here, we identified subsets of cTfh cells that involved in the generation of *P*. *vivax***-**specific MBC and antibody responses. We found that *P*. *vivax* infection induced an expansion of cTfh2 cells. The cTfh2 cell responses were positively related to an increased frequency of activated and atypical MBCs, and plasma cells, as well as *P*. *vivax***-**specific antibody levels. Moreover, phenotype of memory cTfh2 cells was changed from CM to EM phase during infection. The alteration of cTfh17**-**EM cells was significantly related to expansion of activated and atypical MBCs, while the presence of cTfh2**-**CM and cTfh17**-**CM cells was positively related to *P*. *vivax***-**specific MBCs and IgM responses. Data from this study suggests a role of cTfh2 cells in contribution to the development of *P*. *vivax***-**specific MBCs and antibody responses.

## Introduction

*Plasmodium vivax* is the second most common cause of malaria, accounting for high rates of morbidity and mortality in many settings [1]. Its unique biological feature is that it hides for a prolonged period in human liver, in the form of dormant hypnozoites. Due to the limitation of vector control and emerging resistance of anti-malarial drugs, better strategies to control and eradicate malaria infection are needed. In this context, an efficacious malaria vaccine could help in decrease parasite burden and thereby reduce clinical malaria and transmission in the communities [2,3]. Currently, four vivax vaccine candidates (*P*. *vivax* Duffy Binding Protein II (PvDBPII), Circumsporozoite Protein (PvCSP), Sexual Stage 25 protein (Pvs25), and Sexual Stage 230 protein (Pvs230)) have been investigated in clinical trials [4–7]. These antigens in combination with adjuvants showed promising potential immunogenicity, safety and protective efficacy. However, the polymorphisms of vaccine candidate results in generation of strain-specific immunity [8]. It is necessary to understand the mechanism of *P*. *vivax* antigens that drives development of cellular and humoral immune responses and design vaccines direct to development of durable and broadly protective antibodies.

It is acknowledged that the establishment of germinal centers (GCs) in secondary lymphoid organs are required for the acquisition of long-lived MBCs or plasma cells [9]. GC function requires help from follicular helper T (Tfh) cells to generate high-affinity antibodies and MBCs responses (i.e., long-lived plasma cells), which together maintain the circulating antibodies required for protection against reinfection [10]. The Tfh cells induced by vaccination and infection are distinguished based on the expression of the chemokine receptor CXCR5, and the inhibitory receptor programmed cell death protein 1 (PD-1) [11]. Human circulating Tfh (cTfh) cells can also be stratified into distinct subsets based on their expression of CXCR3 and CCR6: CXCR3^+^CCR6^-^ (Th1 type), CXCR3^-^CCR6^+^ (Th17 type), and CXCR3^-^CCR6^-^ (Th2 type) [12]. These Tfh cell subsets appear to have various functions in antibody induction depending on the disease, pathogen and vaccination setting [13].

Although the important role Tfh cells are reported to have in antibody induction, published studies on their function in malaria infection are scarce. Studies in both *P*. *falciparum* and *P*. *vivax* malaria show an increase in the frequency of cTfh cells [14–17]. However, the specific subsets of cTfh cells induced by malaria infection is unclear. Previous studies show an increase of both CXCR3^+^ cTh1 [17] and CXCR3^+^CCR6^-^ cTfh1 phenotypes [14,18] in *P*. *falciparum-*infected children residing in areas of high malaria transmission. Human volunteers with experimental *P*. *falciparum* infection showed an expansion of cTfh2 cells during early infection, while cTfh1 cells become activated after 1 week of following [11]. In *P*. *vivax* malaria, an elevation of the cTfh2 subset of memory CD4^+^ cells was reported in symptomatic Brazilian patients [16], while other studies showed an increase in CXCR3^+^Tbet^+^ cTfh1 cells [19]. An association between cTfh cells and humoral immunity was demonstrated in *P*. *vivax* malaria based on the following: a high frequency of both activated (CD21^-^CD27^+^) and atypical (CD21^-^CD27^-^) MBCs were found in the same subjects who had increased cTfh cells [16], and others have found a positive correlation between frequency of total cTfh cells and anti-merozoite antibody titers [15]. Together, previous studies indicate that malaria infection induces cTfh cells expansion and activation, and that these responses are positively related to antibody levels. However, a gap remains as to which specific Tfh subsets drive the acquisition and persistence of malaria-specific antibody and MBC responses.

Knowledge of the cooperative function between Tfh and B cells has relevance to understanding the acquisition and persistence of humoral immune responses following *P*. *vivax* infection [20]. The heterogeneity of Tfh cells, which are composed of phenotypically distinct subsets, allows for discrete roles in mediating protective immunity. Since the distribution of cTfh subsets in natural *P*. *vivax* infection was reported only in malaria endemic areas of Brazil [15,16] and Indonesia [19], we hypothesized that individuals living in malaria endemic regions with differing transmission intensities and the inflammatory or cytokine milieu induced by *P*. *vivax* infection might lead to differential responses of Tfh cell subsets [21]. Therefore, interactions of certain subsets of *P*. *vivax*-polarized Tfh cells and activated B cells might influence the production of protective *P*. *vivax-*specific MBCs and antibodies, as well as the development of memory Tfh cells. To assess this hypothesis, we comprehensively determined the phenotype of cTfh cell subsets, and identified associations among cTfh cell subsets, MBC subsets, *P*. *vivax*-specific MBCs and antibody responses in *P*. *vivax*-infected patients in a low transmission intensity setting. The kinetic responses of cTfh cell subsets, memory cTfh cells, MBCs and antibodies were monitored in a cohort study. Our data allow a better understanding of the functional role of cTfh cells in driving development of antibody and memory B cell responses to *P*. *vivax* infection.

## Methods

### Ethics statement

This study was approved by the Committee on Human Rights Related to Human Experimentation, Mahidol University Central Institutional Review Board (MU-CIRB 2021/281.2505). Written informed consent was obtained from each participant before blood collection. All experiments involving human subjects were conducted in accordance with relevant guidelines and regulations.

### Study design and participants

Heparinized blood samples were taken from subjects in malaria low-transmission areas in the southern part of Thailand (Chumphon, Ranong and Kanchanaburi provinces). Both *P*. *falciparum* and *P*. *vivax* were common in these endemic areas. Thirty-one *P*. *vivax-*infected patients were recruited for blood collection at the Ministry of Public Health malaria clinics (Vector Borne Disease Units 11.4.2 and 11.5.3) from May 2023 to September 2023. The criteria for recruitment of vivax malaria subjects were: (i) systolic blood pressure greater than 90 mmHg, (ii) body temperature lower than 40°C, (iii) hematocrit higher than 25%, and (iv) age 18 years or higher. Individuals who did not meet these criteria were excluded. Blood samples were taken to determine the frequency and characteristics of cTfh cells and their subsets. To determine correlations between cTfh and humoral responses, the levels of antibodies (total IgM and IgG, and IgG subclasses), frequency of MBCs specific to *P*. *vivax* vaccine antigen (PvDBPII), and MBC subset phenotypes in these acute *P*. *vivax* patients were assessed. Of the 31 acute-phase subjects, 23 who had a high frequency of activated and atypical MBC populations than median + IQR of healthy controls (HCs) were analyzed further to detect expression of T-cell co-stimulatory molecules (CXCR5, IL-21 receptor (IL-21R) and CD40) on cell surfaces. To evaluate the kinetics of cTfh cell responses, MBC and anti-*P*. *vivax* antibody responses, 13 subjects who had high frequencies of cTfh2 and atypical MBCs were monitored at Day 14 and 60 after infection. Acute symptomatic *P*. *vivax* infections were each documented microscopically using both thin and thick Giemsa-stained blood smears, and then confirmed by nested PCR [22]. Thirteen treated subjects, in a 60-day cohort study, were visited at home weekly by malaria clinic staff to estimate the incidence of clinical malaria infections during the study period. Venous blood samples were collected in heparinized vacutainer tubes (Becton Dickinson) and transported to the laboratory within 4–6 h. Data on past malaria infections were obtained from the records kept by the local malaria clinic and Vector Borne Unit 11.4. Sixteen healthy controls (HCs) who were non-malaria infected and lived in non-endemic areas (Bangkok, Thailand) were enrolled to determine control values of cTfh cell and MBC frequencies, and anti-*P*. *vivax* antibody titers. The demographic information of recruited subjects is summarized in **S1 Table.**

### Recombinant protein production

Recombinant PvDBPII and PvCSP were produced following previous study [8,23]. Briefly, DNA sequences encoding PvDBPII and PvCSP were amplified by PCR. The PCR products were cloned into pET23a+ vectors and expressed in *E*. *coli* BL21(DE3) LysE (Invitrogen). Bacterial cultures were induced by 1.0 mM of IPTG for 4 h at 37°C. The expressed inclusion bodies were solubilized, purified and refolded, as previously described [23]. Finally, refolded proteins were purified by HisTrap column and ion exchange chromatography (Cytiva) and the purity was confirmed by SDS-PAGE.

### Flow cytometric analysis

To determine the frequency of cTfh cells in peripheral blood of *P*. *vivax*-infected subjects and healthy controls, cryopreserved PBMCs were thawed and aliquots with >95% viability were used for flow cytometric analysis. After washing, the cells were incubated with Zombie red viability dye (1:400 dilution, Biolegend) for 20 min, followed by further washing in PBS supplemented with 2% FBS. They were then incubated for 20 minutes on ice, in two steps (with two washes in between) using antibodies specific to cTfh or B cell surface antigens. The following antibodies (all from Biolegend) were used for T cell surface staining: CD3-Alexa Flour 700 (1:100), CD4-PerCP-Cy5.5 (1:200), CXCR5-PE (1:50), PD-1-APC/Fire750 (1:50), CXCR3-APC (1:50), CCR6-PE/Cy7 (1:20), ICOS-AF488 (1:50), CD45RA-PE/Dazzle (1:100) and CCR7-FITC (1:50). The following antibodies (all from Biolegend) were used for B cell surface staining: CD19-FITC (1:200), CD38-Alexa Flour 700 (1:200), CD21-APC (1:100), CD27-APC/FIRE (1:50), IgM-PE (1:200), IgD-Pe/Cy7 (1:200), IgG-PerCP-Cy5.5 (1:20), CXCR5-PE (1:200), IL-21R-PE/Cy7 (1:10), CD40-PE/Cy5 (1:25). After staining, the cells were washed twice in PBS with 2% FBS before acquisition by FACSCanto II (BD Bioscience); FACS data were examined using FlowJo software version 10.10.0 and presented as cell percentages or mean fluorescence intensity (MFI). Representative gating strategies for cTfh and B cells phenotyping are shown in **S1 and S2 Figs**.

### Total IgM and IgG ELISA

Anti-PvCSP and -PvDBPII antibody levels in plasma samples were measured using indirect ELISAs, as previously reported [8,23]. Briefly, 96-well microtiter plates were coated with 2 μg/ml recombinant PvCSP or PvDBPII protein and incubated overnight at 4°C. After blocking with 1% FBS in the 1xPBS buffer, plasma samples (diluted 1:200) were added to duplicate wells and incubated for 1 h. After washing, HRP-conjugated goat anti-human IgG or IgM antibody (Seracare) was added into the wells, and incubated for 1 h. Subsequently, TMB (Millipore) was added to detect antigen-antibody reactivity. A Multiscan SkyHigh Microplate Reader (Thermo Scientific) was used to determine the absorbance at 450 nm. The levels of total IgM and IgG specific to PvCSP and PvDBPII were standardized as a reactivity index (RI), calculated by dividing OD values of tested samples by a cut-off value (mean + 2SD) based on HC samples. An RI greater than or equal to 1.0 was considered positive for specific antibodies; an RI less than 1.0 was considered as seronegative responders. On each plate, two acute plasma samples from *P*. *vivax-*infected subjects with high titers from our previous study were included as positive controls; plasma from two HCs were used as negative controls [23]. The experiments were performed in duplicate and the average values were taken for further analysis.

### IgG subclass ELISA

To detect IgG subclass responses to PvCSP and PvDBPII, we used a protocol previously described [23]. Plasma samples of *P*. *vivax-*infected subjects were added at a dilution of 1:100 and HRP-conjugated goat anti-mouse IgG (Biolegend) was added at dilutions of 1:500 for IgG1 and IgG3, and at 1:1000 IgG2 and IgG4, antibodies. Signal was developed with TMB substrate (Millipore) and OD values were read at 450 nm. Two samples with anti-PvCSP and -PvDBPII total IgG responses were used as positive controls. The levels of IgG subclasses specific to PvCSP or PvDBPII were standardized as a reactivity index (RI), calculated by dividing OD values of tested samples by a cut- off value (mean + 2SD) determined in HC samples (n = 16). Positivity of RI values was defined as in IgM and IgG ELISA. Experiments were performed in duplicate and average values were used for analyses.

### ELISPOT

The presence of PvCSP or PvDBPII-specific MBCs was determined by ELISPOT assay as performed in a previous study [8,24]. Briefly, 1 × 10^6^ cells per ml for each antigen were stimulated with a cocktail of R848 (Invivogen) and recombinant human IL-2 (PrepoTech) at 37°C in a 5% CO_2_ incubator for 96 h. The ELISPOT assay was performed by coating with 15 μg/ml of anti-human monoclonal IgG antibodies (clone MT91/145; Mabtech), 5 μg/ml of recombinant PvCSP or PvDBPII, or 1 μg/ml of tetanus toxoid (TT) antigen (Merck Millipore) onto ELISPOT plates (Merck Millipore). The stimulated PBMCs were seeded into duplicate wells. Then, 1 μg/ml of monoclonal antibody MT78/145 (Mabtech) was added and incubated for 2 h. Following thorough washing, diluted (1:1000) streptavidin-HRP-conjugated, polyclonal, goat anti-human IgG (Mabtech) and TMB substrate (Mabtech) were added. The plates were analyzed with a CTL ELISPOT Reader. PvCSP or PvDBPII-specific MBCs were quantified as spot-forming units (SFUs) in the wells. PBMCs of *P*. *vivax*-infected subjects were cultured without stimulators and incubated overnight with antigens were used as negative controls. A positive response of antigen-specific MBCs was defined as more spot-forming cells (SFCs) than twice the number of spots of the negative controls.

### Data analysis

Statistical analyses were performed using GraphPad Prism software version 8.4.3, (GraphPad Software, USA, https://www.graphpad.com) and R version 4.1.1. The non-parametric Mann-Whitney test was used to compare between the results from *P*. *vivax-*infected subjects and healthy controls, and for comparisons among different time points in an individual subject. Multiple group comparisons among MBC populations were compared using Kruskal-Wallis test and Dunn’s multiple comparison test. Wilcoxon matched-pairs signed rank test was used to compare the significant difference between two time points. Statistical significance was considered as p<0.05. For correlation analyses, corrplot package in R version 0.90 was used to plot the graph, and data was clustered by ward.D2 hierarchical agglomerative clustering method [25].

## Results

### Expansion of total cTfh cells and alteration of cTfh cell subsets in *P*. *vivax* malaria

**Fig 1:**
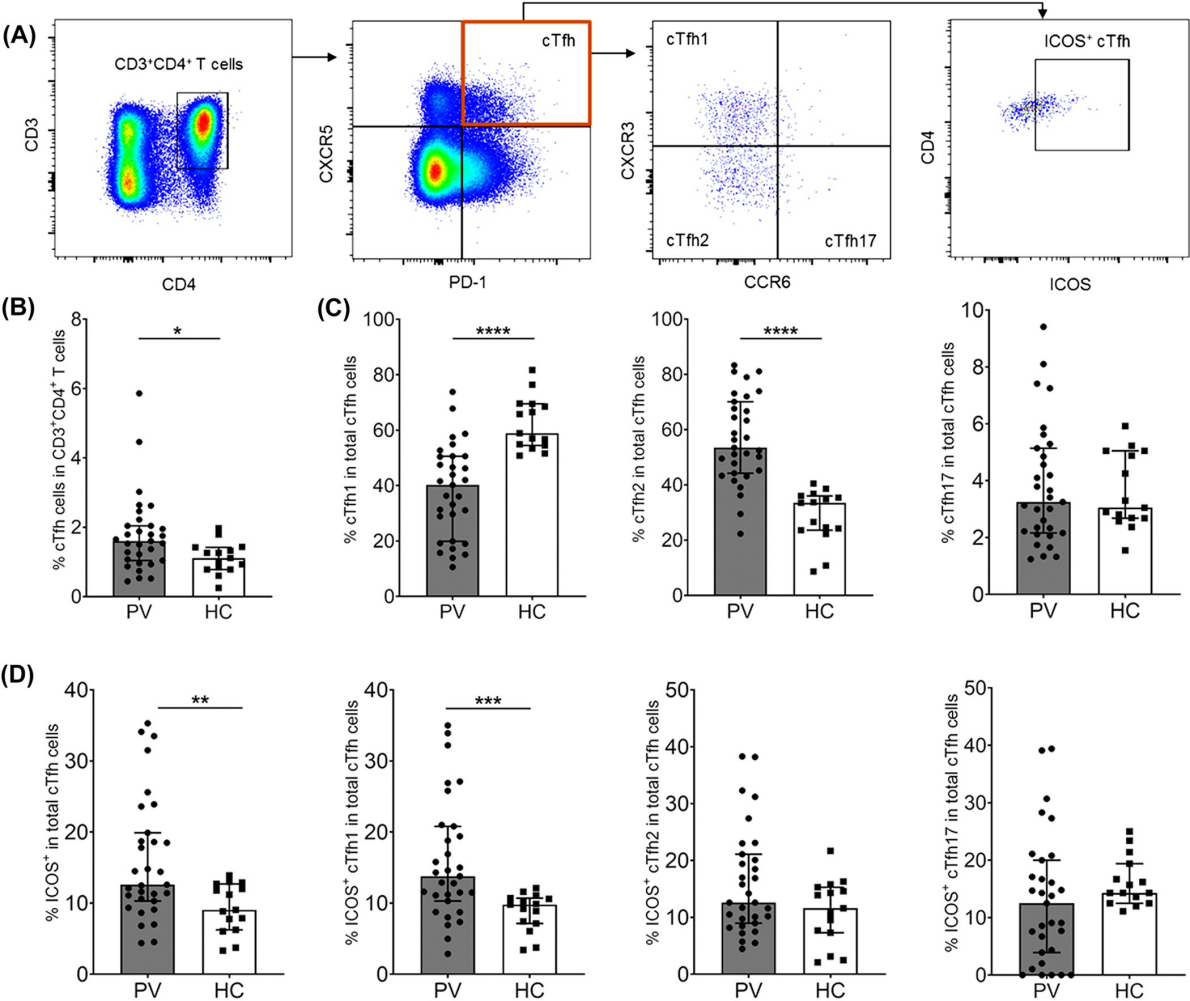
Expansion of total cTfh cells and alteration of cTfh cell subsets in *P*. *vivax* infection. PBMCs from acutely *P*. *vivax* infected patients (PV; n = 31) and healthy controls (HC; n = 15) were phenotyped by flow cytometry. **(A)** Representative gating strategy for circulating cTfh (CXCR5^+^PD-1^+^), cTfh subsets based on the expression of CXCR3, CCR6 (cTfh1; CXCX3^+^CCR6^-^, cTfh2; CXCR3^-^CCR6^-^ and cTfh17; CXCR3^-^CCR6^+^), as well as activation marker (ICOS). **(B)** The frequency of cTfh in CD3^+^CD4^+^ T cells. **(C)** The frequency of cTfh1, cTfh2 and cTfh17 in total cTfh cells. **(D)** The frequency of ICOS^+^ cTfh cells and their subsets in total of cTfh population. Each bar represents median and error bar represents interquartile range (IQR). Statistical testing was performed by Mann-Whitney rank test for comparing two non-parametric groups; * p<0.05, ** p<0.005, ***p<0.0005, **** p<0.0001.

To assess the responses of cTfh cells and their subsets during *P*. *vivax* infection, we quantified cTfh cell activation and subset distribution by flow cytometry in acutely infected patients. The population of cTfh cells was identified by the expression of CXCR5^+^ and PD-1^+^ in CD3^+^CD4^+^ T cells; their subsets were based on the expression of CCR6 and CXCR3 (**Fig 1A**). A significant increase of total cTfh cells in acute *P*. *vivax* patients was detected, as compared to that in HCs (median 1.60 [IQR 1.04–2.04] vs. median 1.11 [IQR 0.78–1.42], p-value<0.05) (**Fig 1B**). Next, we determined the distribution of cTfh subsets in infected individuals and HCs. An expansion of cTfh2 cells was detected in *P*. *vivax* patients compared with HCs (median 53.50 [IQR 44.2–70.1] vs median 33.5 [IQR 23.6–36]), p-value<0.0001), whereas cTfh17 cells were not significantly different (median 3.25 [IQR 10.3–19.9] vs median 9.06 [IQR 6.25–12.7], p-value>0.05). A reduction of cTfh1 cells was observed in *P*. *vivax* subjects (median 40.2 [IQR 19.9–50.6] vs median 58.9 [IQR 54.5–69.5], p-value<0.0001) (**Fig 1C**).

Next, we observed the expression of inducible T cell co-stimulator (ICOS) as an activation marker on cTfh cell subsets of *P*. *vivax* patients, compared to baseline of HCs. ICOS upregulation was greatly detected on the cTfh1 cells of patients (median 13.8 [IQR 10.3–20.8] vs median 9.80 [IQR 7.14–10.7], p-value<0.005) (**Fig 1D**). In contrast, cTfh2 and cTfh17 cells did not significantly differ in their ICOS expression in *P*. *vivax* patients and HCs (cTfh2; median 12.6 [IQR 8.98–21.1] vs median 11.6 [IQR 7.3–15.3], respectively; p-value>0.05; cTfh17; median 12.5 [IQR 3.92–20] vs median 14.3 [IQR 12.5–19.4], respectively; p-value>0.05). There were ten (32.2%, 10/31) and eight (25.8%, 8/31) subjects who had ICOS expression above the HC baselines for cTfh2 and cTfh17, respectively (**Fig 1D**). The relationship between age (> 18 years old) and frequency of cTfh cell subsets was assessed in these *P*. *vivax* subjects and no significant correlation was found (**S3 Fig**).

### Activated and atypical MBCs were predominant responses in *P*. *vivax*-infected individuals

**Fig 2:**
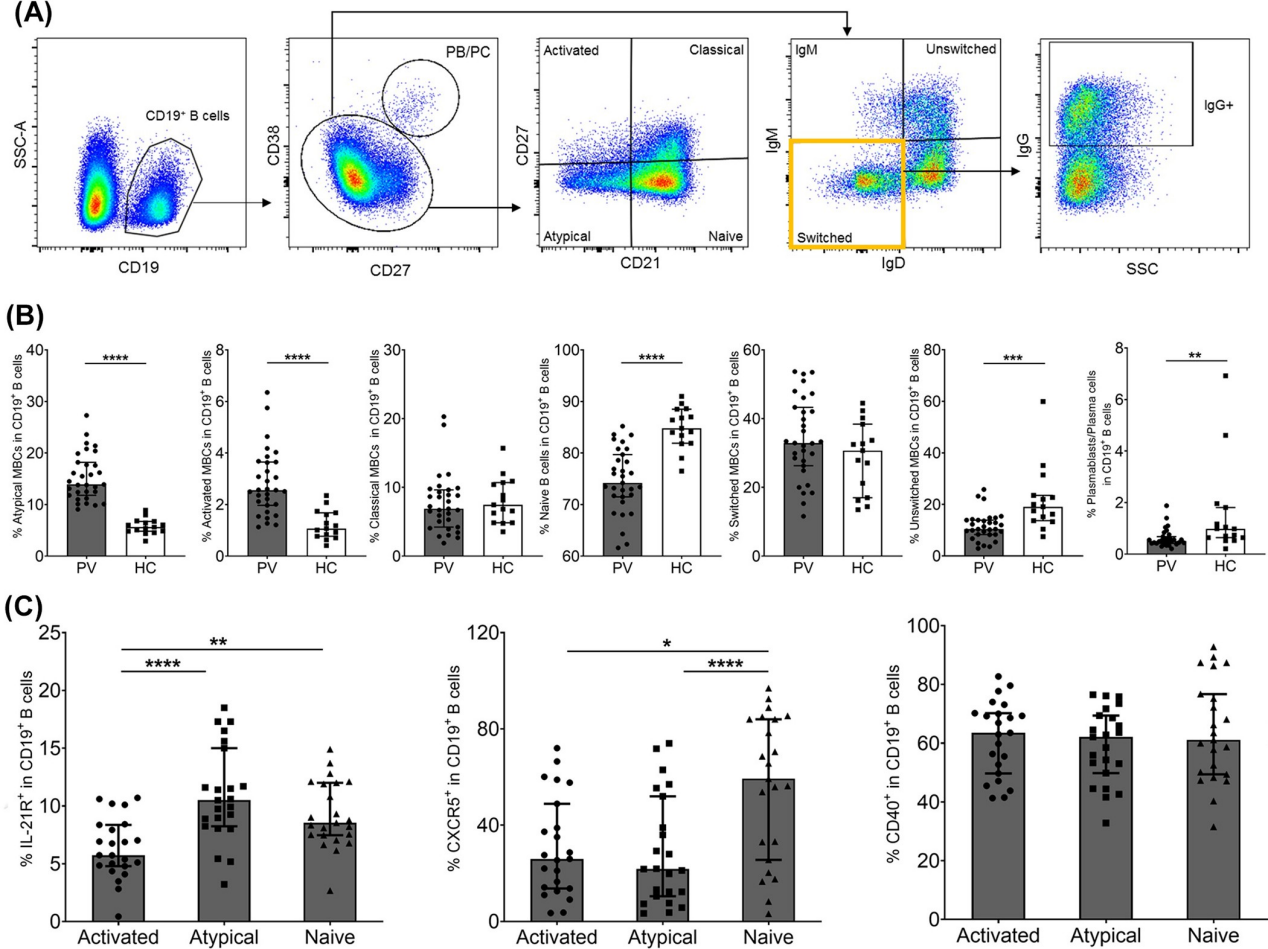
Frequency of each MBC subset during acute *P*. *vivax* infection. PBMCs from acutely *P*. *vivax* infected patients (PV; n = 31) and healthy controls (HC; n = 15) were phenotyped by flow cytometry. **(A)** Representative gating strategy for MBC subsets, including activated MBCs (CD21^-^CD27^+^), classical MBCs (CD21^+^CD27^+^), naive B cells (CD21^+^CD27^-^), atypical MBCs (CD21^-^CD27^-^) and class-switching phenotypes, including unswitched (IgD^+^IgM^+^), and switched (IgM^+^ or IgG^+^) B cells. **(B)** The frequency percentages of MBC subsets present in acutely *P*. *vivax* infected subjects and HCs. An acutely *P*. *vivax* infected patients (n = 23) who showed higher frequency of activated MBCs and atypical MBCs above the median + IQR of HCs. **(C)** The frequencies of CXCR5, IL-21R and CD40 expressing CD19^+^ B cell subsets. Each bar represents median and error bar represents interquartile range (IQR). Statistical testing was performed by Mann-Whitney rank test for comparing two non-parametric groups, and three groups were compared by Kruskal-wallis test with Dunn’s multiple comparisons test; * p<0.05, ** p<0.005, ***p<0.0005, **** p<0.0001.

An increase of atypical MBCs was previously reported in acute *P*. *vivax* subjects living in the same endemic areas as in the present study [23]. However, whether that expansion of atypical MBCs was related to cTfh cell responses was unknown. Here, we identified the MBC subsets that showed increased responses to *P*. *vivax* infection (**Fig 2A**). The frequencies of activated MBCs (median 2.56 [IQR 1.97–3.65] vs. median 1.07 [IQR 0.77–1.68], p-value<0.0001) and atypical MBCs (median 13.9 [IQR 11.8–18.2] vs. median 5.63 [IQR 4.83–6.73], p-value<0.0001) were higher in *P*. *vivax*-infected subjects compared to those in HCs (**Fig 2B**). However, the frequencies of naive B cells (median 74.20 [IQR 71.5–79.7] vs. median 84.8 [IQR 81.9–88.5], p-value<0.001), unswitched MBCs (median 32.9 [IQR 26.3–43.3] vs. median 30.7 [IQR 17.0–38.4], p-value<0.005) and plasmablast/plasma cells (median 0.51 [IQR 0.42–0.68 vs. median 0.98 [IQR 0.64–1.8], p-value<0.05) in acute *P*. *vivax* subjects was lower than HCs (**Fig 2B**). The percentage of classical (median 6.89 [IQR 4.22–9.64] vs. median 7.46 [IQR 4.85–10.7], p-value>0.05), and switched MBCs (median 32.9 [IQR 26.3–43.3] vs. median 30.7 [IQR 17.0–38.4], p-value>0.05) in *P*. *vivax*-infected individuals was not different from those in HCs (**Fig 2B**).

Since Tfh cells produce IL-21 cytokine, which drives the growth and differentiation of B cells and isotype switching [11], we detected the expression of T cell co-stimulatory molecules (CXCR5, IL-21R, and CD40) on the surface of activated and atypical MBCs compared to naive B cells. Thus, twenty-three *P*. *vivax* subjects with frequencies of activated and atypical MBCs above the median + IQR of HCs were selected for further analysis. A significantly higher IL-21R expression was shown in atypical MBCs. The CXCR5 expression was highly detected in naive B cells compared to activated and atypical MBCs (**Fig 2C**). For CD40 molecule, there was no difference in expression between naive and MBCs (activated and atypical) (**Fig 2C**).

### Seropositivity of *P*. *vivax*-specific IgM and IgG responses in individuals with cTfh2 cell expansion

**Fig 3:**
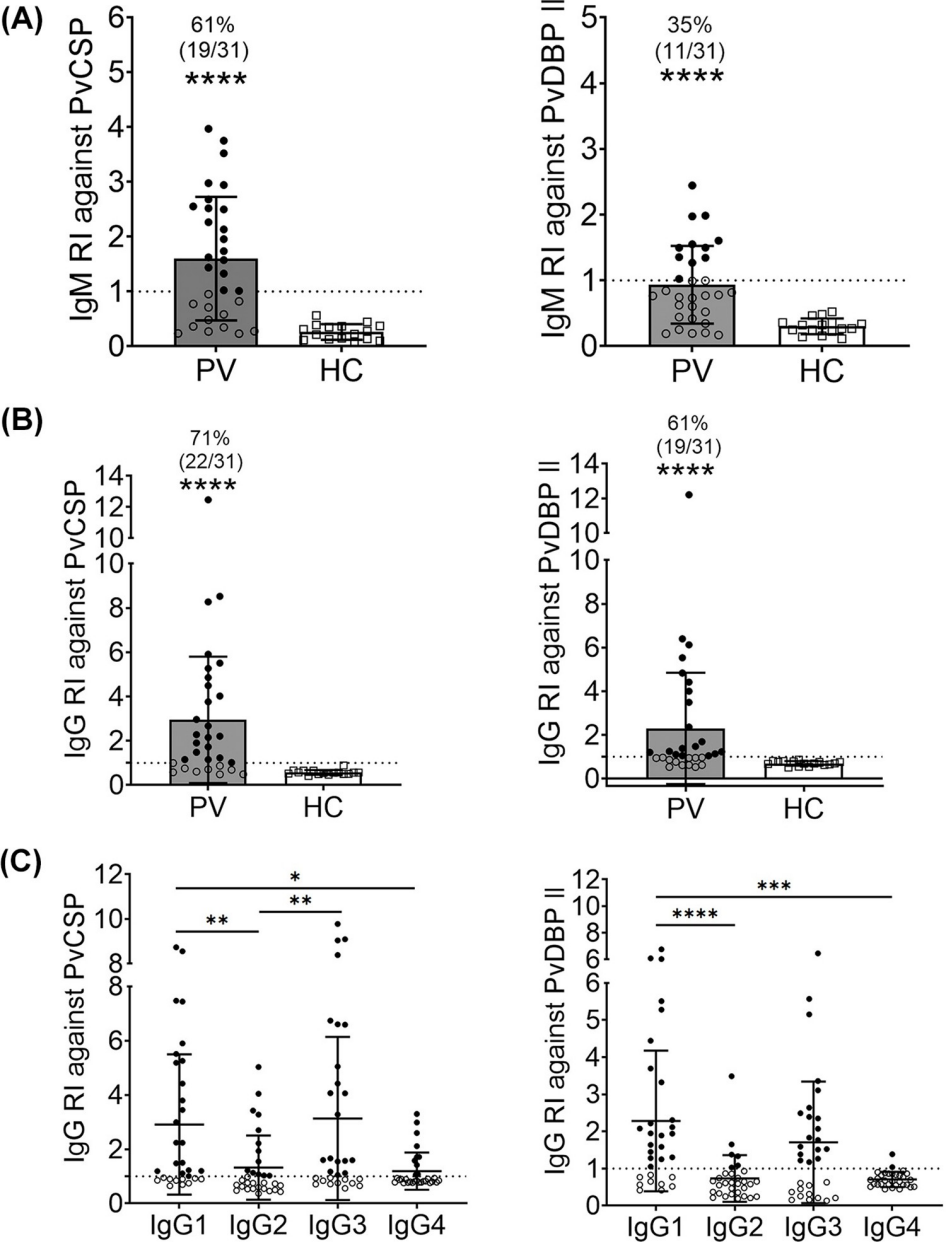
Antibody responses against two *P*. *vivax* antigens (PvCSP and PvDBPII). Reactivity index values (RIs) were determined by using plasma of acutely infected subjects (PV; n = 31) compared to malaria naive healthy controls (HC; n = 16). **(A)** IgM reactivity index (RI) against PvCSP and PvDBPII. **(B)** IgG reactivity index (RI) against PvCSP and PvDBPII. **(C)** IgG subclass (IgG1-4) responses against PvCSP and PvDBPII. Percentages of seropositivity of antibody responses were indicated on the top of scatter plot. Dashed line represents cut-off values for seropositivity (RI = 1) calculated from mean of RI + 2SD of healthy control samples (n = 16). Statistical testing was performed by Mann-Whitney rank test for comparing two non-parametric groups; * p<0.05, ** p<0.005, **** p<0.0001.

To relate an expansion of Tfh with antibody responses, anti-PvCSP and -PvDBPII IgM, IgG and IgG subclass antibody levels were determined in 31 *P*. *vivax-*infected individuals and compared to those in HCs. The percentages with anti-PvCSP and -PvDBPII IgM antibodies were 61 and 35%, respectively (**Fig 3A**). Seropositive IgG responses against these two vaccine candidates were 71% (22/31) to PvCSP and 61% (19/31) to PvDBPII (**Fig 3B**). Analysis of IgG subclass responses to PvCSP and PvDBPII showed that the predominant responses were by the cytophilic IgG1 and IgG3 subclasses (**Fig 3C**). Seropositivities of IgG1, IgG2, IgG3 and IgG4 were 68% (21/31), 42% (13/31), 65% (20/31) and 45% (14/31) against PvCSP, and 71% (22/31), 19% (6/31), 61% (19/31) and 6% (2/31) against PvDBP II, respectively (**Fig 3C**).

### Expansion of cTfh2 cells was positively related to atypical MBCs, while ICOS^+^ cTfh2 cells were positively correlated with plasma cells frequency and antibody levels

**Fig 4:**
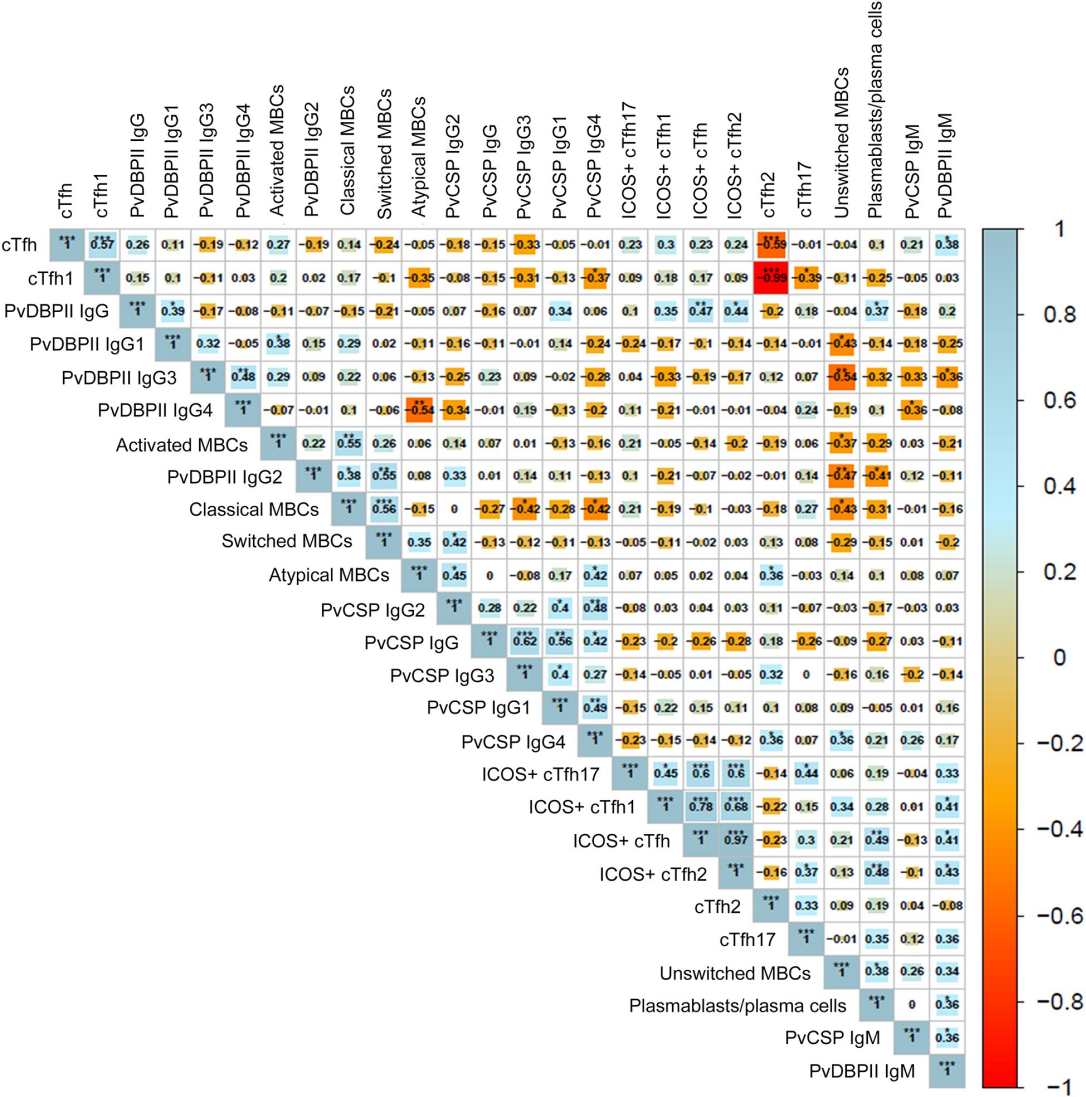
Correlation analysis of cTfh cell subset responses and anti-*P*. *vivax* antibodies. Shown are Spearman’s ρ and significance adjusted for multiple testing using the Holm method. * p<0.05; ** p<0.01; *** p<0.001. Spearman ρ>0.60 and adjusted p-value<0.01 are indicative of a strong positive and highly significant correlation. The coloring of cells represents the sign of correlation: blue gradient for positive correlation and red gradient for negative correlation. The size of background squares represents degree of correlation corresponding to the values shown in each cell.

To determine the relation between an altered proportion of cTfh subsets on humoral immunity, we analyzed the relationships of cTfh2 cell number to MBC subsets and antibody levels in infected individuals. A moderate correlation was defined as a Spearman’s ρ in the range of 0.4–0.6 [24], a strong correlation by a ρ greater than 0.6, and a low correlation by a ρ below 0.4. We observed significant positive correlations between total cTfh cells and PvDBPII-specific IgM (ρ = 0.38, p<0.05), and between cTfh2 cells and both atypical MBCs (ρ = 0.36, p<0.05) and anti-PvCSP IgG4 levels (ρ = 0.36, p<0.05) (**Fig 4**). In regard to ICOS expression, total ICOS^+^ cTfh cells were positively correlated with plasmablasts/plasma cells (ρ = 0.49, p<0.01), PvDBPII-specific IgM levels (ρ = 0.41, p<0.05), and PvDBPII-specific IgG levels (ρ = 0.47, p<0.01) (**Fig 4**). A moderate correlation was also observed between frequency of ICOS^+^ cTfh1 cells and PvDBPII-specific IgM levels (ρ = 0.41, p<0.05), as well as ICOS^+^ cTfh2 cells with PvDBPII-specific IgM (ρ = 0.43, p<0.05), PvDBPII-specific IgG (ρ = 0.44, p<0.05) and plasmablasts/plasma cells (ρ = 0.48, p<0.01) (**Fig 4**).

### PvDBPII-specific MBCs were markedly detected in *P*. *vivax*-infected individuals with cTfh2 expansion

**Fig 5:**
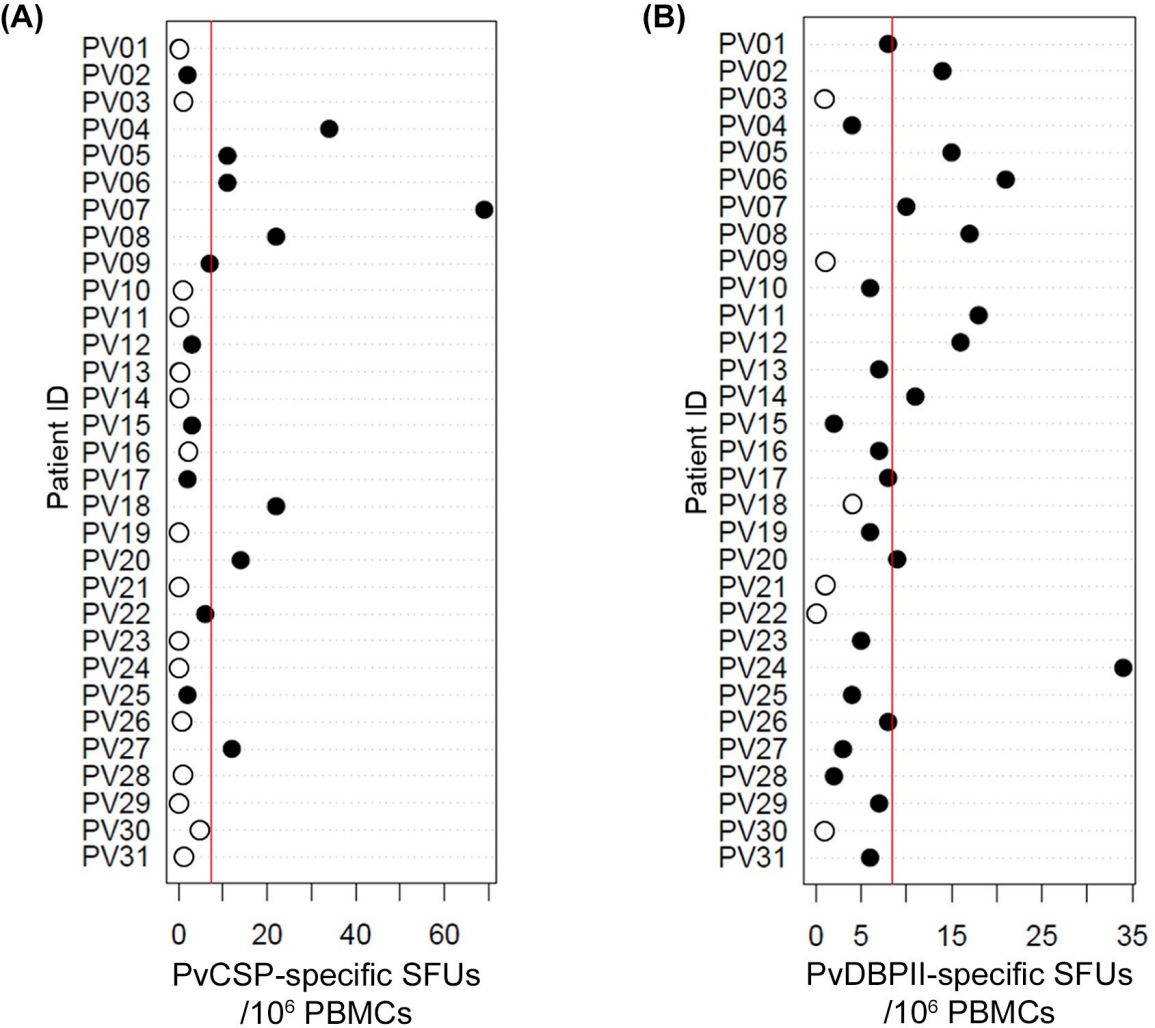
Memory B cell responses against PvCSP and PvDBPII in *P*. *vivax* infected subjects. Spot forming units (SFUs) per 10^6^ PBMCs against two *P*. *vivax* antigens (PvCSP and PvDBPII). The vertical red line represents mean of SFUs of studied *P*. *vivax* infected subjects (n = 31). The black dot represents positive *P*. *vivax* antigen-specific MBC response. The positive response of antigen-specific MBCs was defined as spot-forming cells (SFCs) with 2-fold higher total number of spots relative to negative control.

Previous studies documented that malaria**-**specific MBCs were developed following natural *P*. *vivax* infection [20,21]. Here, we analyzed whether *P*. *vivax* subjects with increased cTfh2 cells produced *P*. *vivax***-**specific MBCs during acute infection. Of the 31 acute patients, the positivity of PvCSP**-** and PvDBPII**-**specific MBCs was 48**% (**15**/**31**)** and 81**% (**25**/**31**)**, respectively. The mean **(**SD**)** number of spots specific to PvCSP was 7.39 **(**14.0**)** and to PvDBPII was 8.48 **(**7.3**) (Fig 5).** We further analyzed PvCSP or PvDBPII specific MBCs in acute *P*. *vivax* subjects **(**n **=** 22**)** who had cTfh2 expansion. The result showed 45.5**% (**10**/**22**)** and 77**% (**17**/**22**)** subjects showed positive PvCSP and PvDBPII**-**specific MBCs, respectively. It was found that 54.5**% (**12**/**22**)** and 23**% (**5**/**22**)** of subjects showed negative PvCSP**-** and PvDBPII**-**specific MBCs **(S2 Table).** All subjects had positive TT**-**specific MBCs **(**mean **[**SD**]**, 38.26 **[**19.3**])** and total IgG MBCs **(**mean **[**SD**]**, 190.55 **[**73.2**])**, not significantly different than HCs **(**mean **[**SD**]**, 161 **[**50.78**]** for total IgG MBCs and 40 **[**16.22**]** for TT**-**specific MBCs**) (S4 Fig).**

### The distribution of memory cTfh cell subsets was altered while the effector phase was mainly represented in cTfh2 and cTfh17 cells

**Fig 6:**
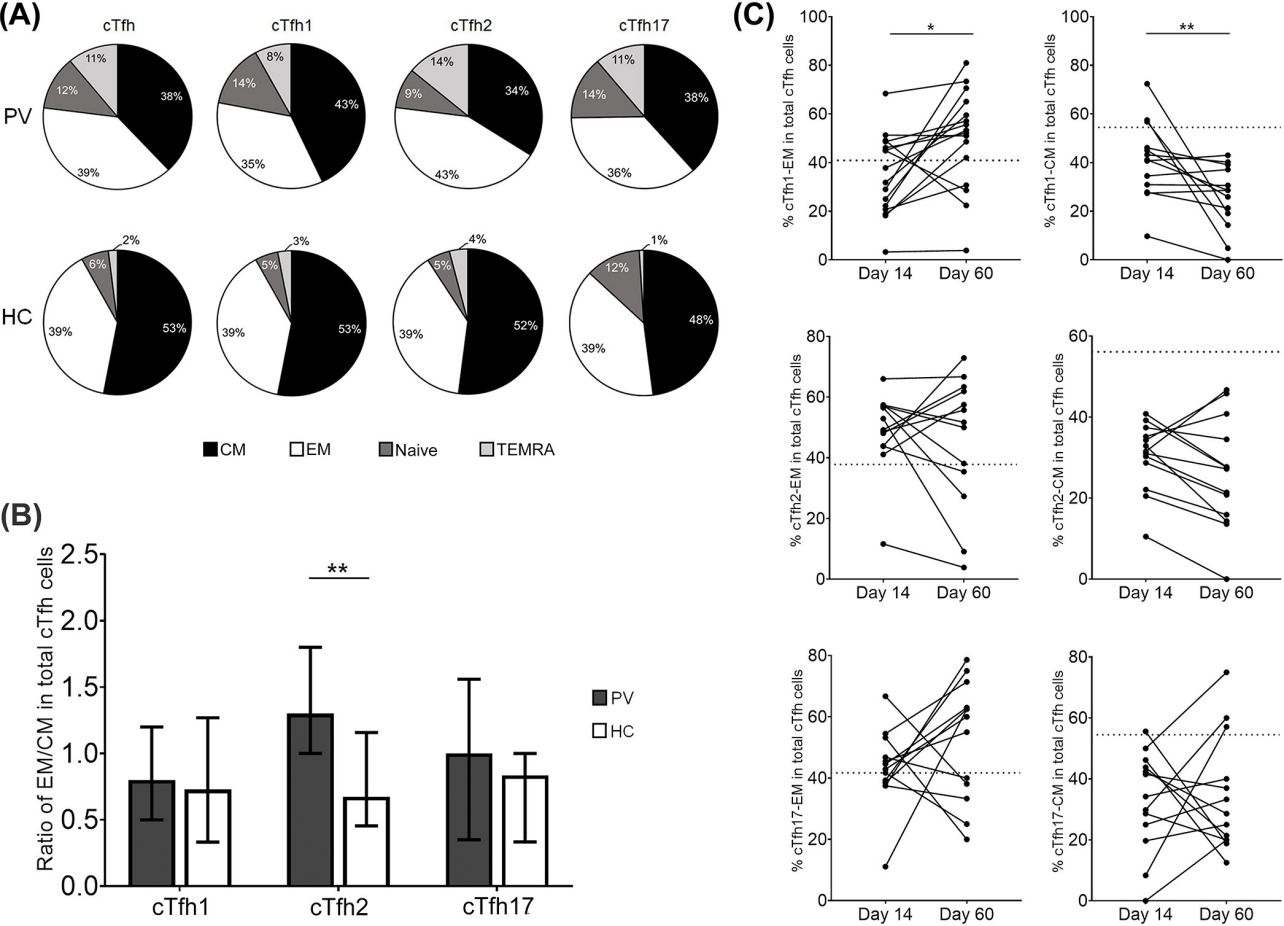
Alteration of memory cTfh phenotypes during *P*. *vivax* infection. **(A)** Distribution of CM and EM phenotypes in total and subsets of cTfh cells in *P*. *vivax* acutely infected subjects (PV; n = 31) and HCs (n = 15). **(B)** Ratio of EM/CM in each cTfh subset of PV compared to HC subjects. Each Bar represents median and error bar represents interquartile range (IQR). A kinetic study was performed in *P*. *vivax* subjects (n = 13) for memory cTfh and subset phenotypes. **(C)** Percentage of EM and CM in each cTfh subset at 2 time points after infection (Day 14 and Day 60). The dashed line represents cut-off values calculated from the median frequency of memory cTfh subset in HCs (n = 7). Statistical analysis for comparison between two groups was performed by Mann-Whitney test: *p<0.05, ** p<0.005. Statistical testing for paired comparison was performed by Wilcoxon matched-pairs signed rank test: * p<0.05 and ** p<0.005. CM; central memory, EM; effector memory, TEMRA; terminally differentiated effector.

Our data showed the development of *P*. *vivax*-specific MBCs during acute infection. To examine whether memory cTfh cells were generated in these subjects, samples from 31 subjects were phenotyped to determine the frequencies of memory cTfh cell subsets (central, effector, TEMRA and naive). During acute malaria, the phenotypes of memory cTfh cell subsets were altered compared to HCs; the predominant subsets found were central memory (CM) and effector memory (EM) cells. The ratio of EM versus CM cells in total cTfh cells was increased in acute *P*. *vivax* subjects (EM/CM = 1.02) compared to the HC ratio (EM/CM = 0.74) (**Fig 6A and 6B**). The memory cell distributions differed among cTfh cell subsets in infected subjects with cTfh2 cells being predominantly by EM cells, while cTfh1 and cTfh17 cells being predominantly by CM cells. A significant increase in EM/CM ratio was shown in cTfh2 compared to HCs (**Fig 6B**). At 60 days post-infection, follow-up responses in 13 individuals showed that most *P*. *vivax* subjects had persistent effector phenotypes, with cTfh2-EM 69% (9/13) and cTfh17-EM cells 46% (6/13) greater than baseline HCs. Significant elevation of cTfh1-EM cell was detected compared to Day 14 (p-value<0.05) (**Fig 6C**). The frequency of CM cells in cTfh1 and cTfh2 subsets was lower than the threshold values in HCs at Day 60, while 3 subjects showed higher cTfh17 cells than baseline HCs. (**Fig 6C**).

### The cTfh17-EM cells were positively related to atypical MBCs, while CM cells in cTfh2 and cTfh17 were associated with *P*. *vivax*-specific MBCs and IgM levels

**Fig 7:**
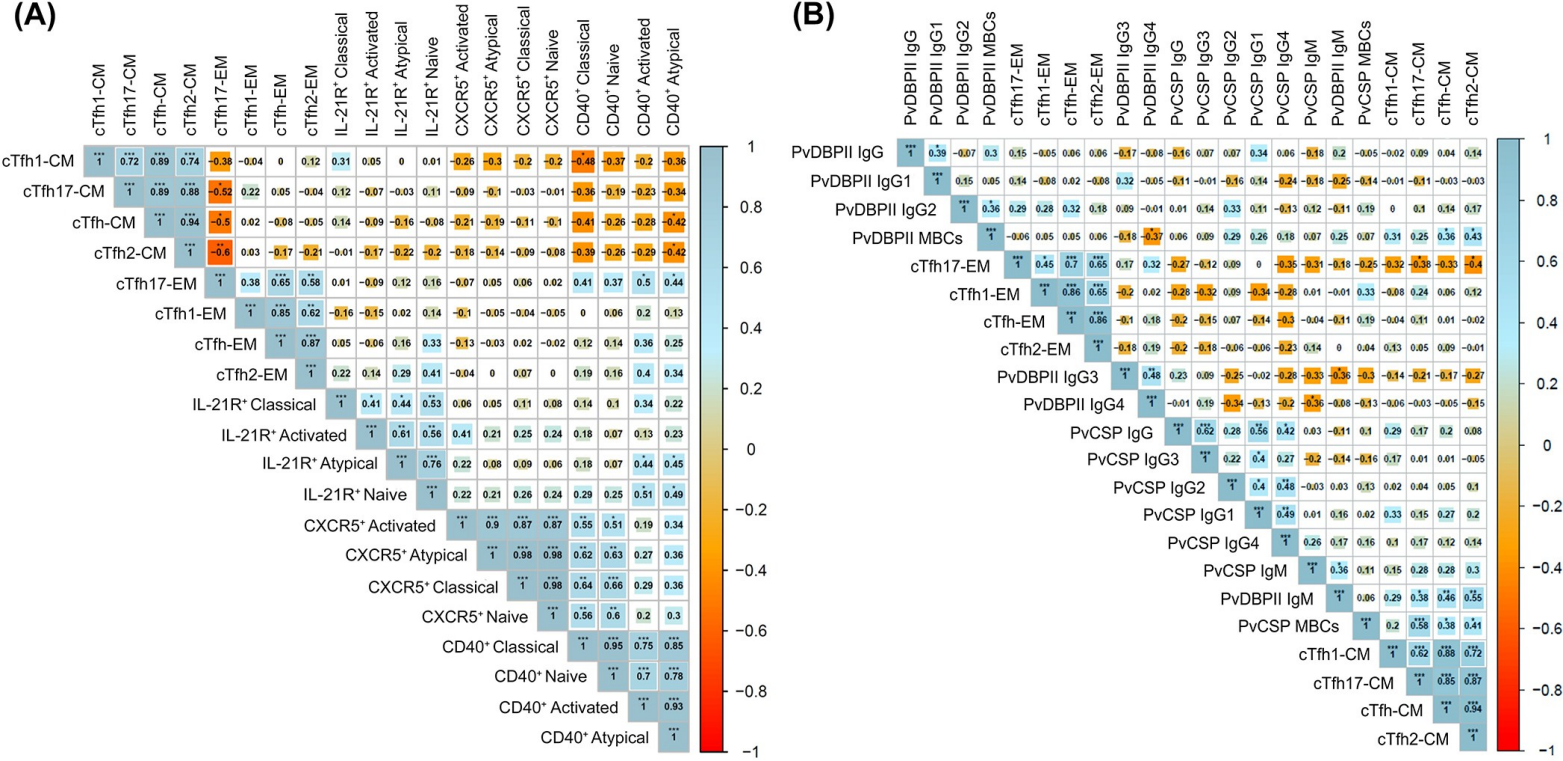
Correlation analysis of memory cTfh cells with anti*-P*. *vivax* humoral immunity. **(A)** Correlation of memory cTfh cells and MBC subsets with overexpression T cell co-stimulatory molecules (CXCR5, CD40, IL-21R) in *P*. *vivax* infected subjects (n = 31). **(B)** Correlation of memory cTfh with *P*. *vivax*-specific MBCs and antibodies (total IgM, IgG and IgG subclasses) in *P*. *vivax* infected subjects (n = 23). Shown are Spearman’s ρ and significance adjusted for multiple testing using the Holm method. * p<0.05; ** p<0.01; *** p<0.001. Spearman ρ>0.60 and adjusted p-value<0.01 are indicative of a strong positive and highly significant correlation. The coloring of cells represents the sign of correlation: blue gradient for positive correlation and red gradient for negative correlation. The size of background squares represents degree of correlation corresponding to the values shown in each cell.

Alterations of memory cTfh cell subsets from CM to EM phenotypes were mainly presented in cTfh2 during acute *P*. *vivax* infection. Thus, we analyzed the relationships of these EM cells with MBC subsets, *P*. *vivax*-specific MBC and antibody responses. The frequency of cTfh17-EM cells was positively correlated with activated and atypical MBCs with CD40 overexpression (ρ = 0.5, p<0.05 and ρ = 0.44, p<0.05) (**Fig 7A**). Next, we assessed whether the changing proportion of memory cTfh cell phenotypes during acute infection was correlated with *P*. *vivax*-specific MBCs and/or antibody responses. The cTfh2-CM cells were found to be positively correlated with the numbers of PvDBPII-specific MBCs (ρ = 0.43, p<0.05) and PvCSP-specific MBCs (ρ = 0.41, p<0.05), as well as anti-PvDBPII IgM levels (ρ = 0.55, p<0.01). Moreover, there were positive correlations between the frequency of cTfh17-CM and both PvCSP-specific MBCs (ρ = 0.58, p<0.001) and anti-PvDBPII IgM levels (ρ = 0.38, p<0.05) (**Fig 7B**). Positive correlations between EM cells in each cTfh subset were found in neither *P*. *vivax*-specific MBCs nor antibody responses in our *P*. *vivax* subjects (**Fig 7B**).

### Kinetic responses of cTfh subsets and humoral immunity in *P*. *vivax* patients

**Fig 8:**
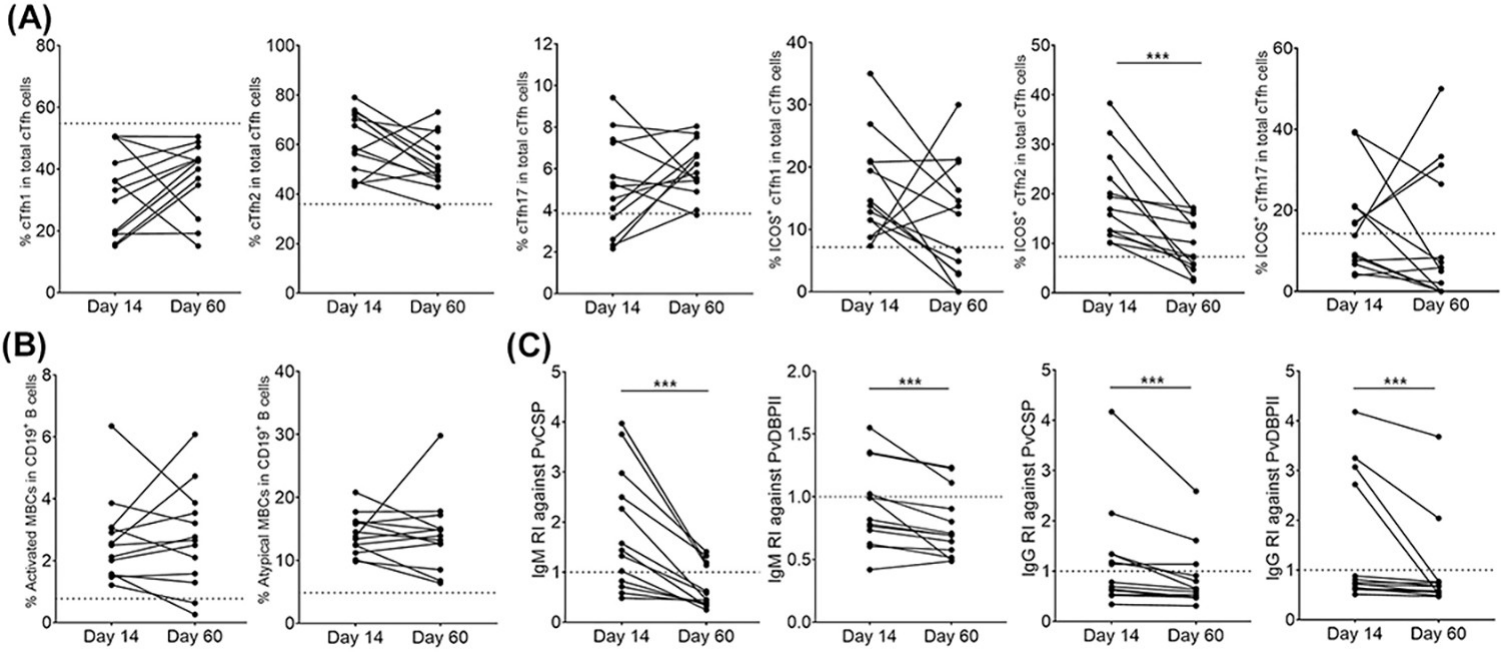
Kinetics of cTfh subsets, MBC subsets and antibodies after recovery from *P*. *vivax* infection. Kinetics study was performed in *P*. *vivax* subjects (n = 13). **(A)** Kinetics of cTfh subsets (cTfh1, cTfh2 and cTfh17) and ICOS expression at Day 14 and Day 60 after recovery. **(B)** Kinetics of MBC subsets (activated and atypical MBCs). **(C)** Kinetics of reactivity index (RI) of total IgM and IgG specific to PvCSP and PvDBPII at Day 14 and Day 60 post-infection. Dashed line represents cut-off values calculated from median of frequencies for cTfh subsets and MBC subset populations detected in HCs (n = 7), and the cut-off value of positive reactivity index (RI = 1) for antibody response. Statistical testing was performed by Wilcoxon matched-pairs signed rank test: *** p<0.0005.

To gain a better understanding of the association between cTfh cell subsets and MBCs that lead to the production of antibodies during *P*. *vivax* infection, we observed kinetic responses in 13 *P*. *vivax* subjects at two time points during the recovery phase (Days 14 and 60). At day 60 post-infection, 92.3% (12/13) and 92.3% (12/13) of subjects maintained the frequency of cTfh2 and cTfh17 cells above baseline values (cTfh2; median 50.0 [IQR 46.30–62.0], cTfh17; median 5.8 [IQR 5.14–7.1]) (**Fig 8A**). Among ICOS^+^ cells, a significant reduction was found in ICOS^+^ cTfh2 cells (Day 14, median 16.90 [IQR 12.15–25.25] vs. Day 60, median 7.4 [IQR 5.16–14.95], p-value<0.0005). Even though some subjects had frequencies below cut-off, there was no significant decrease in overall frequency of ICOS^+^ cTfh1 nor ICOS^+^ cTfh17: ICOS^+^ cTfh1 (Day 14, median 13.80 [IQR 10.13–20.90] vs. Day 60, median 12.5 [IQR 2.88–18.55]) and ICOS^+^ cTfh17 (Day 14, median 13.8 [IQR 7.15–20.95] vs. Day 60, median 5.8 [IQR 0.0–28.85]) (**Fig 8A**). In follow-up of MBC subsets at 60 days post-infection, the frequency of activated MBCs (85%, 11/13) and atypical MBCs (100%, 13/13) were consistently increased above the baseline values of HCs (**Fig 8B**). At day 60 post-infection, significant decreases were observed in IgM and IgG responses to PvCSP and PvDBPII antigens. Four subjects (30.7%, 4/13) maintained positive IgM responses to PvCSP and three (23%, 3/13) to PvDBPII. For IgG responses, three subjects (23%, 3/13) were positive for PvCSP and two (15.3%, 2/13) for PvDBPII (**Fig 8C**).

## Discussion

For malaria vaccines to be protective requires that they induce long-term antibody and MBC responses. Tfh cells play a role in driving activated B cells into GCs for generation of high-affinity antibodies, MBCs and long-lived plasma cells (LLPCs) [20]. Given the several known subsets or phenotypes of Tfh cells (Tfh1, Tfh2 and Tfh17), more details of the function of each Tfh cell subset for helping B cell activation and generation of humoral immunity are required. Here, the response of cTfh cells to *P*. *vivax* infection was demonstrated in low malaria transmission areas of Thailand, with very few of our subjects having a history of prior infection, based on records of malaria clinics. We found that activation of cTfh cells by *P*. *vivax* infection led to altered proportions of Tfh cell subsets towards Tfh2 cells (CXCR3^-^CCR6^-^) in circulating blood. An expansion of this cTfh2 cell phenotype (by 58% of subjects as shown by ICOS overexpression) was similar to findings in a Brazilian study of *P*. *vivax* infections that showed an increased frequency of ICOS^hi^PD1^+^CXCR5^+^CD4^+^ cTfh2 cells in the memory CD4^+^ T cells population [16]. However, these results contrasted with those of a study by Figueiredo and colleagues that showed no alteration in the proportion of cTfh cells within memory CD4^+^ T cells during acute illness [16]. To gain more knowledge of the relationship between cTfh2 cells and humoral immunity, we determined correlations between cTfh cells, MBC subsets and antibodies. In 31 *P*. *vivax* subjects, 71 and 61% were seropositive for anti-PvCSP and -PvDBPII IgG antibodies, while 61 and 35% were positive for anti-PvCSP and anti-PvDBPII IgM antibodies, respectively. The percentage of ICOS^+^ cTfh2 cells was positively correlated between frequency of plasmablasts/plasma cells (ρ = 0.48) and anti-PvDBPII-IgM and IgG titers (ρ = 0.43, and ρ = 0.44, respectively). The data represented here demonstrated that *P*. *vivax* infection altered the proportions of cTfh cell subsets toward cTfh2, and the activation of this cell subset was correlated with development of antibody responses. However, our observation also found that there was a decreased frequency of cTfh1 cells. Significantly increased frequency of ICOS^+^ cTfh1 was positively related to anti-PvDBPII IgM levels. This result might be explained by the migration of activated ICOS^+^ cTfh1 cells from circulating blood to peripheral tissues or lymphoid organs upon *P*. *vivax* infection. It will be interesting to observe the function of cTfh1 in inducing IgM responses in future studies.

An expansion of atypical MBCs was detected in both *P*. *falciparum* and *P*. *vivax* patients [26,27]. However, it is unclear whether these cells require interaction with Tfh cells as part of the mechanism producing anti-malarial antibodies. There is limited data in this domain of human malaria. In our study, *P*. *vivax* subjects who had increased cTfh2 cells showed high frequency of activated (CD21^-^CD27^+^) and atypical (CD21^-^CD27^-^) MBCs in their CD19^+^ B cell populations. There were positive correlations between the frequency of cTfh2 cells and atypical MBCs (ρ = 0.36) and anti-PvCSP IgG4 levels (ρ = 0.36). However, cTfh1 and cTfh17 subsets did not show correlation to atypical MBCs. Our data support the previous studies in function of cTfh cells for helping atypical MBCs differentiation into antibody-secreting cells. A study of *P*. *falciparum* infections showed that atypical MBCs differentiated into ASCs when co-cultured with autologous Tfh cells from malaria*-*exposed individuals [28]. In addition, a recent study in *P*. *vivax* malaria subjects demonstrated a synergistic effect of several molecular signals (IFN-γ, IL-21 and TLR7/8 ligands) in promoting the differentiation of atypical MBCs into ASCs and antibody production [26]. Together, these data indicate that there is cooperation between Tfh cells and atypical MBCs in producing anti-malarial antibodies. Future studies will need to assess the ability of Tfh2 cell subset to drive malaria-specific atypical MBCs development and differentiation into ASCs, likely required for malaria vaccine efficacy.

Alterations in the distribution of cTfh cell subsets were described in both falciparum and vivax malaria [11,14,16–18,29]. The cTfh1 cell phenotype is more prevalent in subjects who live in high malaria transmission areas. *P*. *falciparum-*infected Malian children have an increase in CXCR3^+^ Tfh1 cells [17], while other *P*. *falciparum*-infected children have high levels of cTfh1 cells (CXCR3^+^CCR6^-^) [14]. *P*. *vivax* infections are associated with elevation of CXCR3^+^PD-1^+^T-bet^+^ Tfh cells [19]. In contrast, human volunteers with experimental *P*. *falciparum* infections [11] and patients with symptomatic *P*. *vivax* infections [15], as well as subjects in this study all had expansions of cTfh2 cells. The differences in Tfh subset responses in malaria might be explained by several host and parasite factors. First, host age may have an effect. One report of acute *P*. *falciparum* infections stated that cTfh cells in adults were higher than those in children [14]. Here, our study determined the frequency of cTfh subsets in *P*. *vivax* infected adults (>18 years old) and the result found no correlations between frequencies of each cTfh subset and age. Second, the duration of parasite infection may have an effect. A previous study of cTfh cell kinetics in subjects of an experimental human challenge with *P*. *falciparum* showed that cTfh2 cells were increased at peak parasitemia, while cTfh1 cells were markedly increased at day 14 after *P*. *falciparum* parasite inoculation [11]. In natural *P*. *vivax* infections, cTfh2 cells were increased and cTfh1 cells decreased during symptomatic malaria. However, in these subjects, alterations in these two subsets persisted after two months. Third, parasite density and/or transmission setting may have an effect. A study reported the positive correlation between activation and proliferation of cTfh2 and parasitemia in natural *P*. *falciparum* infection [14]. In *P*. *vivax* infection, it was demonstrated that the number of malaria episodes was positively correlated with Tfh frequency [16]. In the present study, cTfh2 cell expansion and parasite density in *P*. *vivax*-infected individuals were not correlated (ρ = 0.04). Fourth, the cytokine condition during malaria infections may have an impact. Inflammatory environments and cytokine milieu can induce Tfh cells to differentiate into different subsets as it was shown in acute falciparum malaria that IFN-γ cytokine responses preferentially activated CXCR3^+^ Tfh cells [17]. Here, the increased frequency of cTfh2 (CXCR3^-^CCR6^-^) cells in acute vivax malaria might be induced by IL-4-producing T helper 2 (Th2) cells, as they have been shown to function in Tfh2 cell differentiation in other infection models [14,21,30]. Further analyses of cytokine secretion profiles of each subset of cTfh and Th cells would be beneficial for better understanding the mechanisms by which *P*. *vivax* infection leads to polarization of cTfh cells.

The development of malaria-specific MBCs following natural infections has been reported [10,24]. However, understanding the persistence of MBCs after infection requires further knowledge to explain: (i) whether malaria infections trigger generation of long-lived MBCs, and (ii) if Tfh cells are required to promote this persistence. In this study, we demonstrated the development of *P*. *vivax*-specific MBCs during acute *P*. *vivax* infections. Among 31 *P*. *vivax-*infected subjects, 15 (48%) and 25 (81%) subjects produced PvCSP- and PvDBPII-specific MBCs, respectively. These data indicate that a blood-stage (PvDBPII) antigen is more immunogenic in the induction of MBCs development than a sporozoite (PvCSP) antigen. Relating the development of *P*. *vivax*-specific MBCs to cTfh cell subsets, the present study found positive correlations among frequency of cTfh2-CM, *P*. *vivax*-specific MBCs and anti-PvDBPII IgM titers. The direct relationship between cTfh2-CM and MBCs indicated that *P*. *vivax* infections altered cTfh2 responses (as shown by the expansion and activation of cTfh2 cells), and that interactions between Tfh and activated B cells led to development of CM cells (in cTfh2 cells) and *P*. *vivax*-specific MBCs. It will be of interest in future studies to investigate the generation of *P*. *vivax-*specific memory Tfh2 cells and the role of Tfh2 cells in supporting persistence of MBCs and/or LLPCs, as well as to observe the stimuli and signals which induce Tfh2-CM cells to differentiate into EM cells. Such observations should be helpful in refining malaria vaccine development strategies.

Here, we observed the distributions of memory Tfh cell phenotypes. An alteration of CM to EM phenotype was detected in *P*. *vivax* patients compared to HCs. The ratio of cTfh-EM/cTfh-CM increased. This finding was in contrast to those observed in human volunteers with experimental *P*. *falciparum* infection who had increased EM and decreased CM Th1-cTfh phenotypes [11]. For the predominant responses of CM phenotype in cTfh17 subset, our data was similar to the previous studies in *P*. *falciparum* malaria-naive adults and Malian children [11,17]. Adding to the knowledge of cTfh17 cell function involved in malaria MBC responses, our data revealed positive correlations among cTfh17-CM, *P*. *vivax*-specific MBCs and anti-PvDBPII IgM levels. Also, cTfh17-EM cells were positively correlated to CD40^+^ classical and atypical MBCs. A possible explanation of these findings is that *P*. *vivax* infection induced cTfh17 cell responses. Accordingly, CM cells in this cTfh subset were differentiated into effector memory cells upon parasite antigen or cytokine triggering during infection. The cytokines (IL-17, IL-21, IL-22 and TNF-α) produced by cTfh17-EM cells could drive classical and atypical MBC activation. These MBCs could differentiate into plasma cells for antibody production [31]. In contrast, no interaction between cTfh2-EM and humoral immunity was observed in this study, suggesting that the different stimuli were involved in the generation of the effector phase of cTfh2 and cTfh17 cells. Deep investigations are needed to better understand the mechanisms of *P*. *vivax*-induced memory cTfh17 development and the differentiation of EM cells in this cTfh cells subset, as well as its function in helping B cells produce antibodies against *P*. *vivax*.

It should be noted that there were several limitations in this study. A positive correlation between cTfh2 and atypical MBCs and its interaction in development of antibodies needs to be confirmed by assessing the frequency of *P*. *vivax* antigen-specific atypical MBCs in infected individuals, as previously reported in *P*. *falciparum* subjects [32]. This study had a small sample size and the number of follow-up time points was limited. A larger sample size and increased durability of post-infection monitoring should help gain better understanding of the functions of each Tfh cell subset in helping development of durable antibody and MBC response to malaria. Of note, *P*. *vivax* subjects in 60-day cohort study were monitored clinical malaria weekly, whereas parasite density was not detected, thus these cohort subjects could not define asymptomatic malaria. Most of our *P*. *vivax* subjects had no history of prior infection, based on the record at malaria clinics. Thus, our study could not demonstrate the correlation between cTfh subsets and the number of prior infection or re-infection. In kinetic study of cTfh cells, MBCs and antibody responses, we did not detect *P*. *vivax* specific MBCs in individual subjects at Day 14 and Day 60 post-infection. Our data could not address an association between cTfh2 cells and durability of PvCSP or PvDBPII-specific MBC response. More knowledge of function of cTfh2 cells in providing help to germinal center (GC) B cells and MBC diffentiation is needed to support the presence of long-lived PvDBPII-specific MBCs up to 3 years after infection [24]. In addition, cytokine measurement was not performed in this study; characterization of cytokine profiles secreted from each subset of cTfh cells and helper T cells of *P*. *vivax*-infected individuals would be helpful in explaining the polarization of cTfh cells by *P*. *vivax* infection. Here we utilized two promising vaccine candidates (pre-erythrocytic PvCSP and blood-stage PvDBPII) to assess possible correlations between antibody levels and frequency of cTfh cell subsets. To advance PvCSP- and PvDBPII-based vaccine design, demonstration of functional antibodies against these two antigens and their association with cTfh cell responses would be very helpful.

## Supporting information

S1 Table Demographic information of *P*. *vivax* subjects and malaria naive healthy donors recruited in this study. (DOCX)

S2 Table The association of cTfh2 expansion with PvCSP- and PvDBPII-specific MBC responses. (DOCX)

S1 Fig Gating strategy for phenotyping cTfh cells and memory cTfh phenotypes. cTfh cells were distinguished by CXCR5 and PD-1. For cTfh cell subsets were distinguished by CXCR3 and CCR6 as cTfh1 (CXCR3^+^CCR6^-^), cTfh2 (CXCR3^-^CCR6^-^), and cTfh17 (CXCR3^-^CCR6^+^). For cTfh cells activation based on ICOS^+^ molecule. Memory cTfh cell subsets were distinguished by CCR7 and CD45RA from total cTfh population as central memory (CM) (CCR7^+^CD45RA^-^), effector memory (EM) (CCR7^-^CD45RA^-^), terminally differentiated effector (TEMRA) (CCR7^-^CD45RA^+^), and naive (CCR7^+^CD45RA^+^) in *P*. *vivax* infected individual (PV).
(TIF)

S2 Fig Gating strategy for phenotyping memory B cell subsets. Memory B cell subsets were distinguished by CD21 and CD27 as activated MBCs (CD21^-^CD27^+^), atypical MBCs (CD21^-^CD27^-^), classical MBCs (CD21^+^CD27^+^), and naive B cells (CD21^+^CD27^-^). Co-stimulatory molecules (CXCR5, IL-21R and CD40) for classical MBC subset were determined in *P*. *vivax* infected individual (PV).
(TIF)

S3 Fig Correlation between age and percentage of cTfh and subsets (cTfh1, cTfh2 and cTfh17). The correlation was performed by Spearman correlation. Straight line represents trend of correlation. Spearman ρ and p-value for each correlation were indicated.
(TIF)

S4 Fig Tetanus toxoid-specific and total IgG MBC ELISPOT results. (A) Numbers of tetanus toxoid (TT) specific- and total IgG spot forming units (SFUs) in million PBMCs of *P*. *vivax* infected subjects (PV, n = 31) in comparison with healthy control subjects (HC, n = 16). The height of bar represents average number of SFUs and error bar represents standard deviation (SD). (B) Representative ELISPOT results showing spot forming units of total IgG MBCs and MBCs specific to tetanus toxoid, PvCSP and PvDBPII of wells without stimulation and wells after stimulation with R848 and IL-2.
(TIF)

S1 File Minimal dataset for Figs 1–8 of this study. (XLSX)
