# Supplementary material for: Polarization toward Tfh2 cell involved in development of MBC and antibody responses against Plasmodium vivax infection
Source: PLoS Negl Trop Dis. 2024 Oct 30;18(10):e0012625. doi: 10.1371/journal.pntd.0012625 (PMC11524495; doi:10.1371/journal.pntd.0012625)
Supplement: S1 Table — (DOCX) [file pntd.0012625.s001.docx]

| **Characteristics** | **Acute**  ***P. vivax*  patients**  **(Day 14)** | **Recovered**  ***P. vivax*  patients**  **(Day 60)** | **Healthy subjects** |
| --- | --- | --- | --- |
| **Total Number** | 31 | 13 | 16 |
| **Age (years)** | | | |
| Median (Q1, Q3) | 37.0 (21.0, 49.0) | 36.7 (22, 45.0) | 25.0 (21.8, 28.0) |
| **Gender** | | | |
| Male | 71% (22/31) | 56% (9/13) | 50% (8/16) |
| Female | 29% (9/31) | 25% (4/13) | 50% (8/16) |
| **Nationality** |  |  |  |
| Thai | 91% (27/31) | 100% (13/13) | 100% (16/16) |
| Myanmar | 9% (4/31) | 0% (0/13) | 0% (0/16) |
| **No. of prior infection** | | | |
| 0 | 30 | 13 | 16 |
| 1 | 1 | 0 | 0 |
| **Parasitemia (parasites/µL)** | | | |
| Mean ± SD  (Range) | 3538.25 ± 2911.98  (360.00 – 9694.00) | 0 | 0 |

**S1 Table. Demographic information of *P. vivax* subjects and malaria naive healthy donors recruited in this study.**
