# Supplementary material for: Polarization toward Tfh2 cell involved in development of MBC and antibody responses against Plasmodium vivax infection
Source: PLoS Negl Trop Dis. 2024 Oct 30;18(10):e0012625. doi: 10.1371/journal.pntd.0012625 (PMC11524495; doi:10.1371/journal.pntd.0012625)
Supplement: S2 Table — (DOCX) [file pntd.0012625.s002.docx]

**S2 Table. The association of cTfh2 expansion with PvCSP- and PvDBPII-specific MBC responses.**

|  |  | **PvCSP-specific MBCs** | | |
| --- | --- | --- | --- | --- |
|  |  | (+) | (-) | Total |
| **cTfh2 cell expansion** | (+) | 10 | 12 | 22 |
|  | (-) | 5 | 4 | 9 |
|  | Total | 15 | 16 | 31 |

|  |  | **PvDBPII-specific MBCs** | | |
| --- | --- | --- | --- | --- |
|  |  | (+) | (-) | Total |
| **cTfh2 cell expansion** | (+) | 17 | 5 | 22 |
|  | (-) | 8 | 1 | 9 |
|  | Total | 25 | 6 | 31 |

The presence of *P. vivax* antigens-specific MBCs was determined by ELISPOT assay. A positive response of antigen-specific MBCs was defined as more spot-forming cells (SFCs) than twice the number of spots of the negative controls. The frequency of cTfh2 was determined by flow cytometry. Both assays were performed by using the PBMCs from acutely infected patients (Pv; n= 31). The expansion of cTfh2 was defined as the frequency percentage higher than median plus IQR of HCs. (+) represents positive results and (-) represents negative results.
