# Supplementary figures and images for: Polarization toward Tfh2 cell involved in development of MBC and antibody responses against Plasmodium vivax infection
Source: PLoS Negl Trop Dis. 2024 Oct 30;18(10):e0012625. doi: 10.1371/journal.pntd.0012625 (PMC11524495; doi:10.1371/journal.pntd.0012625)

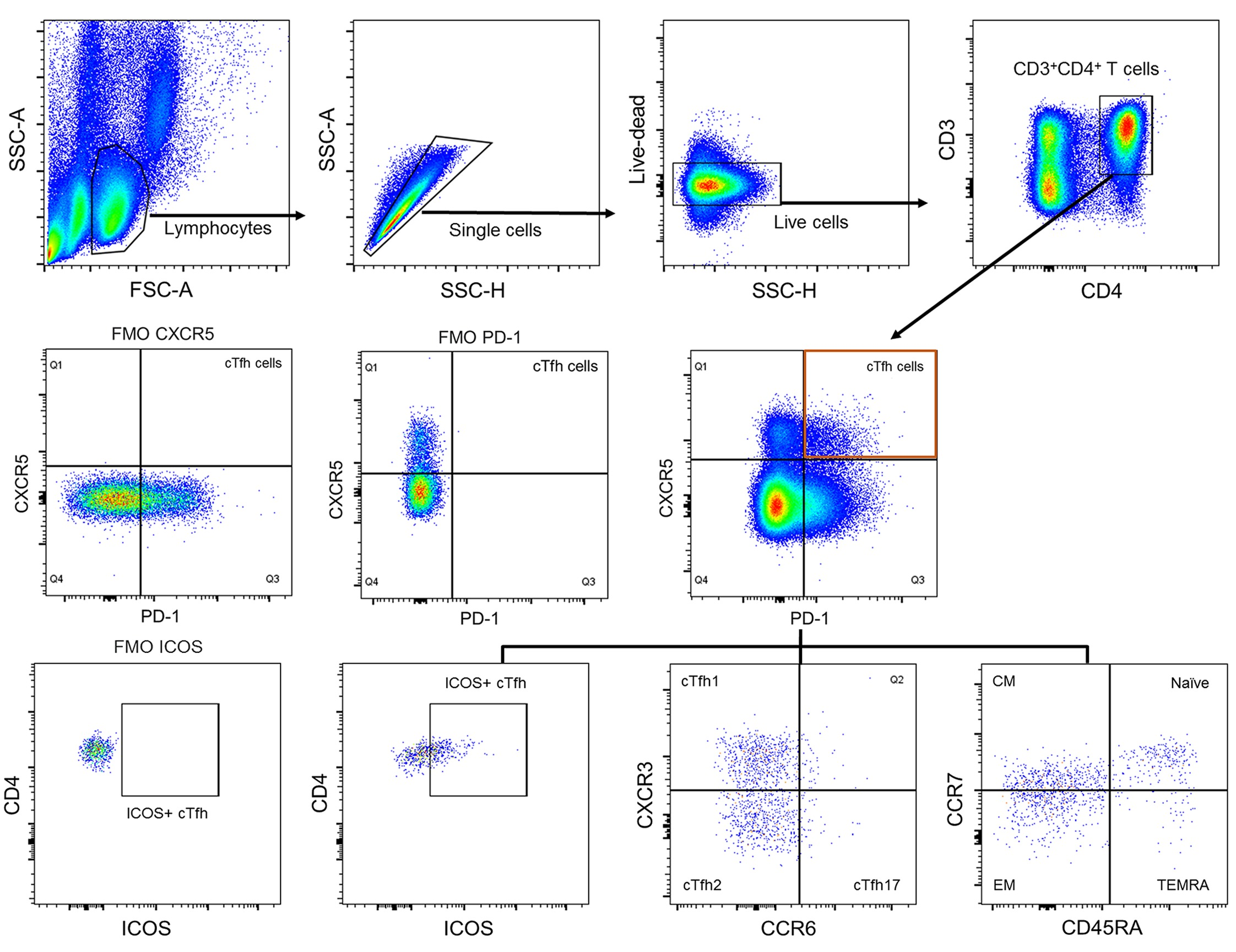

Supplement: S1 Fig — cTfh cells were distinguished by CXCR5 and PD-1. For cTfh cell subsets were distinguished by CXCR3 and CCR6 as cTfh1 (CXCR3+CCR6-), cTfh2 (CXCR3-CCR6-), and cTfh17 (CXCR3-CCR6+). For cTfh cells activation based on ICOS+ molecule. Memory cTfh cell subsets were distinguished by CCR7 and CD45RA from total cTfh population as central memory (CM) (CCR7+CD45RA-), effector memory (EM) (CCR7-CD45RA-), terminally differentiated effector (TEMRA) (CCR7-CD45RA+), and naive (CCR7+CD45RA+) in P. vivax infected individual (PV). (TIF) [file pntd.0012625.s003.tif]

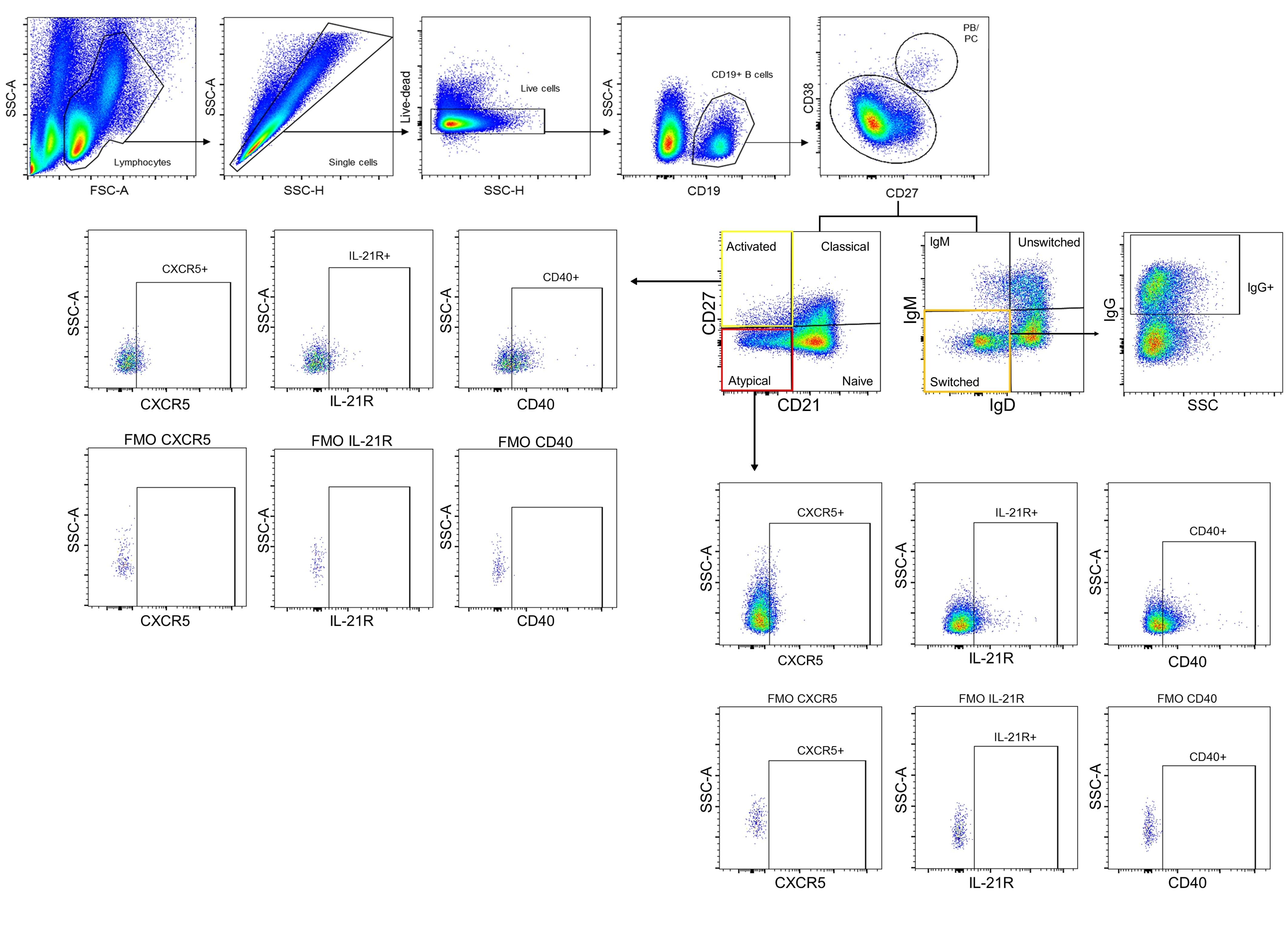

Supplement: S2 Fig — Memory B cell subsets were distinguished by CD21 and CD27 as activated MBCs (CD21-CD27+), atypical MBCs (CD21-CD27-), classical MBCs (CD21+CD27+), and naive B cells (CD21+CD27-). Co-stimulatory molecules (CXCR5, IL-21R and CD40) for classical MBC subset were determined in P. vivax infected individual (PV). (TIF) [file pntd.0012625.s004.tif]

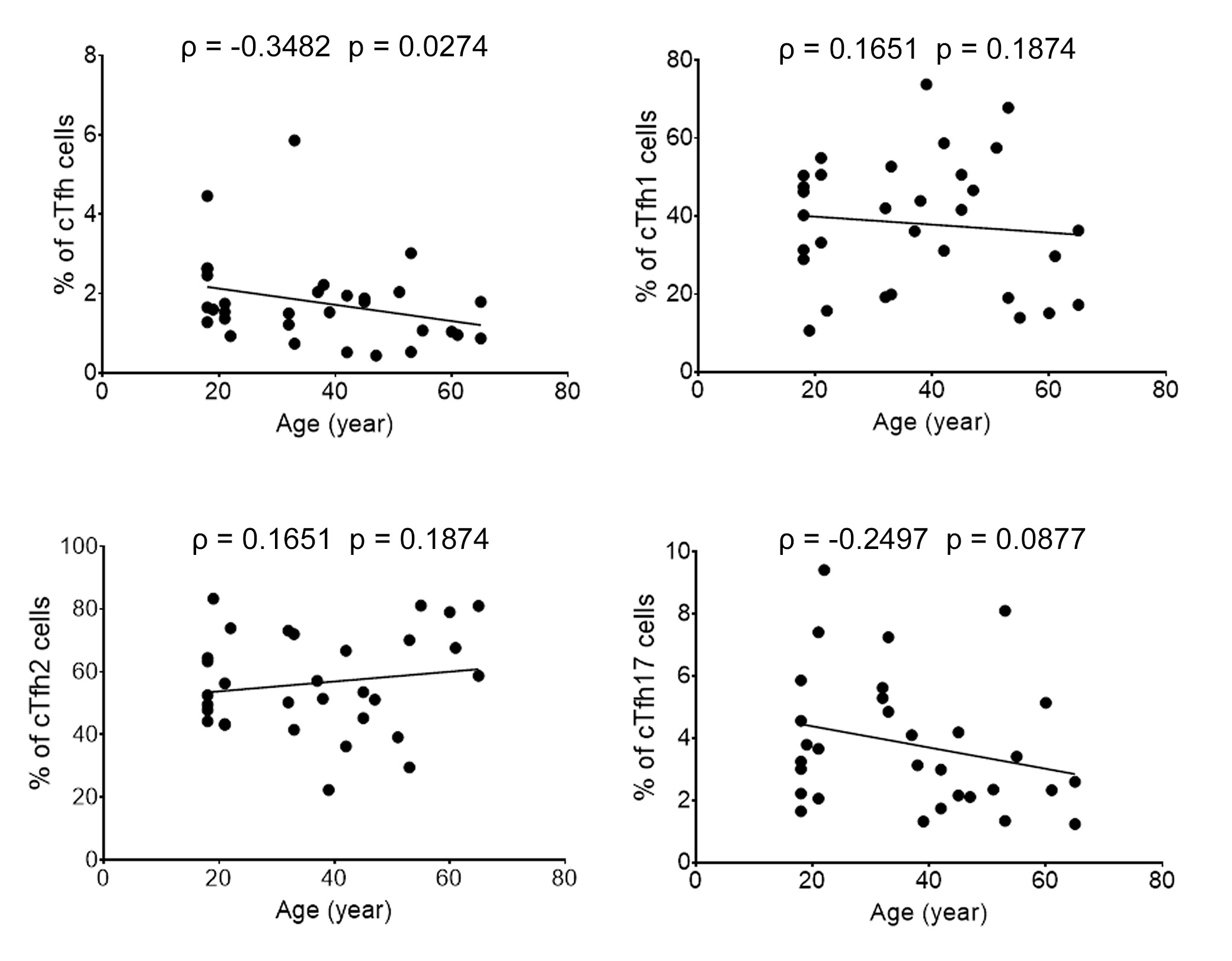

Supplement: S3 Fig — The correlation was performed by Spearman correlation. Straight line represents trend of correlation. Spearman ρ and p-value for each correlation were indicated. (TIF) [file pntd.0012625.s005.tif]

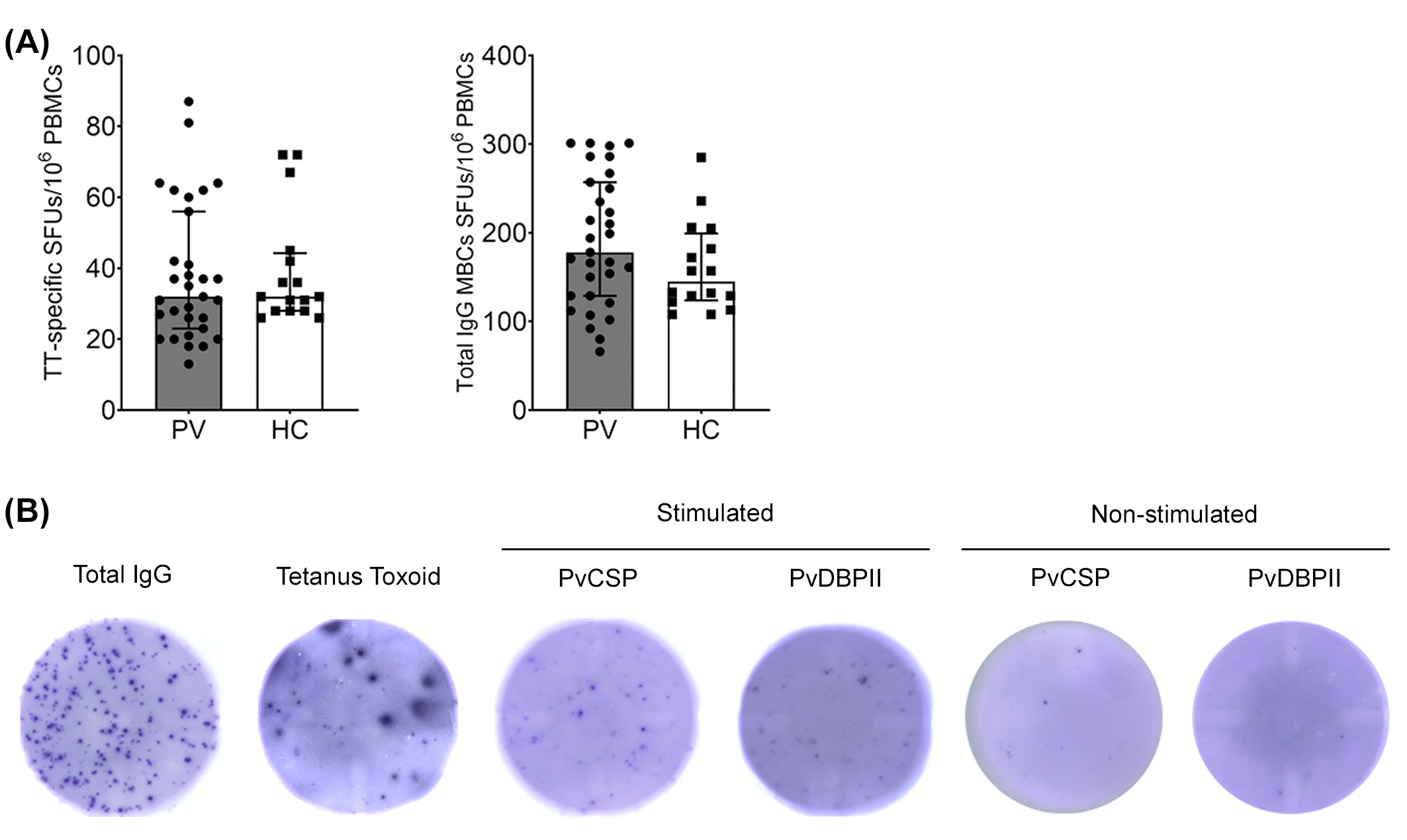

Supplement: S4 Fig — (A) Numbers of tetanus toxoid (TT) specific- and total IgG spot forming units (SFUs) in million PBMCs of P. vivax infected subjects (PV, n = 31) in comparison with healthy control subjects (HC, n = 16). The height of bar represents average number of SFUs and error bar represents standard deviation (SD). (B) Representative ELISPOT results showing spot forming units of total IgG MBCs and MBCs specific to tetanus toxoid, PvCSP and PvDBPII of wells without stimulation and wells after stimulation with R848 and IL-2. (TIF) [file pntd.0012625.s006.tif]
